# Early warning of postoperative recurrence in trigeminal neuralgia: a systematic review and meta-analysis of prediction models

**DOI:** 10.3389/fneur.2026.1772331

**Published:** 2026-05-07

**Authors:** Xinxin Tian, Mingpeng Shi, Guohui Zhou, Yueliang Sun, Yuqing Shi, Huazhong Xiong, Mengchen Wang, Jiaxin Dong, Jixiang Ren

**Affiliations:** 1 College of Traditional Chinese Medicine, Changchun University of Chinese Medicine, Changchun, China; 2 Department of Pain, Affiliated Hospital of Changchun University of Chinese Medicine, Changchun, China; 3 College of Integrated Chinese and Western Medicine, Changchun University of Chinese Medicine, Changchun, China; 4 Prevention and Treatment Center, Affiliated Hospital to Changchun University of Chinese Medicine, Changchun, China

**Keywords:** meta-analysis, predictive learning models, recurrence, systematic review, trigeminal neuralgia

## Abstract

**Background:**

Postoperative recurrence remains a major challenge in trigeminal neuralgia surgery. Prediction models are crucial for personalized management, but their quality and performance are unclear.

**Methods:**

We searched eight databases up to September 23, 2025, for studies on trigeminal neuralgia recurrence prediction. Data extraction followed the CHARMS checklist, and risk of bias was assessed using the Prediction Model Risk of Bias Assessment Tool. A random-effects model was used to meta-analyze the area under the curve, with subgroup and sensitivity analyses.

**Results:**

Twenty studies (4,291 patients) were included. The pooled area under the curve was 0.86 for the training set and 0.83 for the validation set. The main sources of bias included inaccuracies in predictor measurement, inconsistent definitions of recurrence, and incomplete reporting. Models based on microvascular decompression appeared to perform best. Key predictors included age 65 years or older, disease duration longer than 5 years, atypical pain, and specific surgical approaches.

**Conclusion:**

This is the first meta-analysis in this field, and suggests that prediction models for trigeminal neuralgia recurrence demonstrate promising discriminatory performance. However, given the potential risks of bias, publication bias, and heterogeneity, the pooled AUC may be overestimated and should therefore be interpreted with caution.

**Systematic review registration:**

https://www.crd.york.ac.uk/PROSPERO/recorddashboard, CRD420251153545.

## 1 Introduction

Trigeminal neuralgia (TN) is a chronic neuropathic disorder characterized by sudden, severe, electric-shock-like facial pain that is often described as one of the most intense pains experienced by humans. This debilitating condition frequently impairs patients’ quality of life, leading to psychological distress and social isolation (1–4). When pharmacological therapy fails to provide adequate relief for patients with trigeminal neuralgia (TN), surgical intervention becomes a key therapeutic option. Commonly employed procedures include microvascular decompression (MVD), percutaneous balloon compression, and radiofrequency thermocoagulation. Numerous studies have demonstrated the efficacy of these approaches in achieving pain relief (5–7). However, postoperative pain recurrence remains a major challenge in TN management. Long-term follow-up studies indicate recurrence rates ranging from approximately 10 to 30%, with an annual risk of 1–4% in many series (7, 8). Such relapses can occur unpredictably, exacerbating patient suffering, increasing healthcare burden, and elevating risks associated with reoperation. Consequently, the early prediction of recurrence risk is of paramount importance for optimizing TN treatment strategies (3, 9).

The advancement of precision medicine has placed increasing emphasis on developing models for the personalized prediction of recurrence risk (10–12). These models integrate clinical features, imaging data, and operative parameters to identify high-risk patients, thereby providing decision support for customizing follow-up protocols and implementing early intervention strategies (13–15). In recent years, there has been a marked increase in research in this domain, resulting in the creation of numerous prediction models that employ either traditional statistical methods (such as logistic regression and Cox proportional hazards regression) or machine learning algorithms (such as random forest) (16). Nonetheless, the majority of these studies are single-center and retrospective, with substantial heterogeneity in predictor selection, outcome definitions, modeling approaches, and validation methodologies. This variability underscores the need for a systematic evaluation of their methodological quality and predictive performance (13, 17). Although these prediction models vary in design and clinical setting, synthesizing their findings through meta-analysis is scientifically justified. Pooling data from heterogeneous studies enhances overall sample size and statistical power, enabling the identification of generalizable predictors and performance patterns that may elude individual investigations. Currently, a comprehensive synthesis, comparative assessment of performance, and consolidation of evidence for these models remain absent, which hinders the selection of the most robust tool and its translation into routine clinical practice (18, 19). A meta-analytic approach, by aggregating data across these diverse studies, can generate more reliable estimates of model performance and delineate consistent predictors, thereby bolstering the evidentiary foundation to inform clinical decision-making.

Therefore, this study aims to systematically assess the discriminatory ability, calibration, and methodological quality of existing predictive models for postoperative recurrence in trigeminal neuralgia (TN) via a systematic review and meta-analysis, while also identifying key predictive factors. This work will guide the development of future high-quality predictive models and, ultimately, facilitate the optimization of postoperative TN management strategies, thereby enhancing long-term patient outcomes.

## 2 Methods

### 2.1 Study design

The present study adhered to the PRISMA guidelines (20) and the CHARMS statement (21). The study protocol was registered in the PROSPERO database (Registration ID: CRD420251153545).

### 2.2 Search strategy

Eight databases were systematically searched: PubMed, Cochrane Library, Embase, Web of Science, China National Knowledge Infrastructure (CNKI), VIP, Wanfang, and SinoMed. The search covered the period from database inception to September 23, 2025, with no language restrictions beyond Chinese and English. The search strategy combined subject headings and free-text terms, primarily including “trigeminal neuralgia,” “percutaneous balloon compression,” “microvascular decompression,” “surgery,” “prediction model,” and “risk assessment.” The full search strategies for all databases are presented in Supplementary material 1.

### 2.3 Inclusion and exclusion criteria

Inclusion criteria: (1) Population: adult patients (aged ≥18 years) with a clinical diagnosis of primary (idiopathic) trigeminal neuralgia who had received surgical treatment; (2) Intervention/Exposure: any surgical intervention for trigeminal neuralgia; (3) Outcome: studies reporting a predictive model for postoperative recurrence of pain (defined as pain reappearance following surgery), with sufficient quantitative data provided for analysis (e.g., C-statistic or equivalent performance metrics); (4) Study design: observational studies (including cohort and case–control designs) or diagnostic accuracy studies focused on the development or validation of a predictive model; and (5) Language: publications in English or Chinese.

Exclusion criteria: (1) case reports, reviews, commentaries, editorials, letters, or other non-original research publications; (2) studies lacking outcome data specific to postoperative pain recurrence; (3) studies that neither developed nor validated a predictive model; and (4) animal studies or non-clinical investigations.

### 2.4 Study selection

Two investigators (XXT and MPS) independently performed the screening process: duplicates were first removed, followed by a sequential assessment of titles, abstracts, and full texts. A third investigator (JXR) was consulted to resolve any disagreements. In addition, the reference lists of the included studies were manually searched for additional relevant records.

### 2.5 Data extraction and quality assessment

In this study, the included prediction models were classified according to the TRIPOD statement (10). Data extraction was performed using the CHARMS checklist (21). The quality of the included prediction models was assessed using PROBAST (Prediction model Risk Of Bias Assessment Tool) (17). PROBAST consists of four domains and 20 signaling questions and is specifically designed to evaluate prognostic prediction models with diverse outcome measures and modeling techniques. Based on the model classification, risk-of-bias and applicability assessments were conducted for each study using PROBAST, with the overall risk of bias rated as low, high, or unclear. Data extraction was conducted independently by two reviewers (YLS and GHZ), with any discrepancies resolved by consensus following discussion with a third reviewer (MCW). Similarly, quality assessment was performed independently by two reviewers (MPS and YLS), with any disagreements resolved through discussion with a third reviewer (XXT).

### 2.6 Statistical analysis

Forest plots were used to display the performance evaluation results of the predictive models. The discriminative performance of each model was quantified using the area under the curve (AUC) and its 95% confidence interval (CI). AUC values range from 0 to 1, with values >0.75 generally considered to indicate good discrimination and values <0.5 indicating poor discrimination. For studies reporting AUC values with 95% CIs, the corresponding standard errors (SEs) were derived from the CIs using the log transformation formula reported by reference (22). Following the recommendations of Debray et al. (19), the AUC values and their SEs were log-transformed before meta-analysis. A random-effects model using restricted maximum likelihood (REML) estimation was employed to pool AUC estimates across individual studies. Forest plots were then generated to visually display the pooled effect sizes (AUC) and their 95% CIs (22). The pooled effect size reflects the average discriminative performance across the combined studies, whereas subgroup-specific forest plots illustrate the influence of particular factors (e.g., surgical approach, scale type, and modeling method) on model performance. All statistical analyses and visualizations were conducted using R version 4.3.1 with the meta package. Between-study heterogeneity was evaluated using the I^2^ statistic and Cochran’s Q test (with *p* < 0.05 denoting statistically significant heterogeneity), whereas subgroup differences were assessed with the χ^2^ test. Model robustness was assessed via sensitivity analyses stratified by training and validation datasets. An additional sensitivity analysis was conducted, restricted to English-language publications, to evaluate whether excluding non-English studies would substantially alter the pooled estimates. The predictive value of key factors was evaluated using forest plots of odds ratios (ORs) for the most frequently reported predictors. The corresponding OR estimates and their 95% CIs were extracted from regression results in the included studies and subsequently pooled via meta-analysis. Publication bias was assessed using funnel plots, with asymmetry evaluated via statistical tests (Egger’s test with corresponding *p*-values).

## 3 Results

### 3.1 Study selection

**Figure 1:**
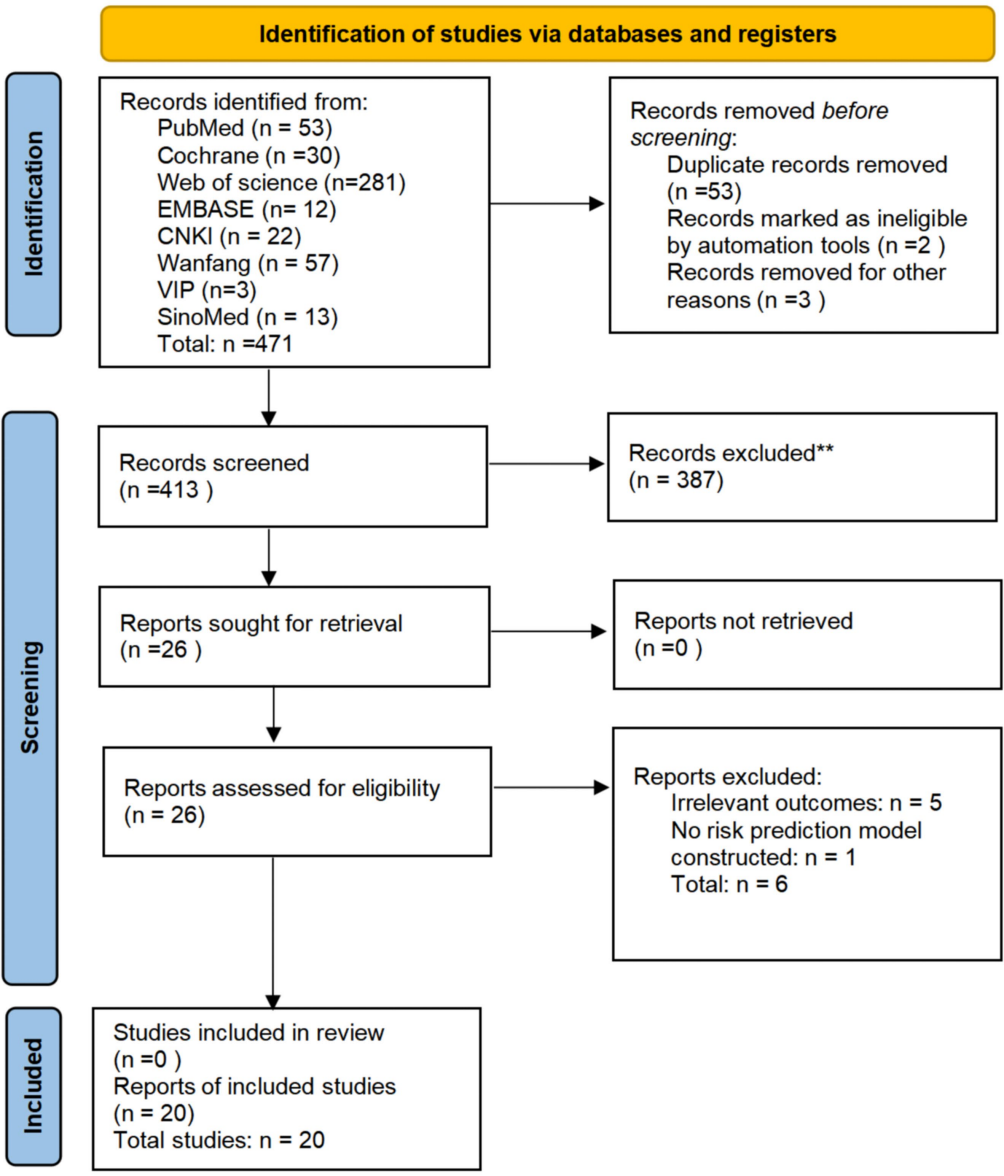
Flowchart of study selection.

The initial database search identified 471 records. After removing duplicates, 413 records remained. Title and abstract screening excluded 387 records, leaving 26 for full-text assessment. Following full-text assessment, 6 studies were excluded, resulting in the inclusion of 20 studies in the systematic review (Figure 1).

### 3.2 Characteristics of the included studies

A total of 20 studies (23–42) were included, encompassing various prediction models for postoperative recurrence of trigeminal neuralgia. The surgical procedures primarily consisted of microvascular decompression (MVD), percutaneous balloon compression (PBC), percutaneous foramen ovale radiofrequency thermocoagulation (RF), and percutaneous radiofrequency thermocoagulation (PRT). The included studies were predominantly conducted in China [19 studies; (23–36, 38–42)], with only one study each from France and the United States (37). Most studies adopted a retrospective design [18 studies; (23–27, 29–39, 41, 42)], while only two were prospective (28, 40). Sample sizes ranged from 56 to 455 cases, yielding a total of 4,291 participants across all studies. Sex distribution varied among studies, patient ages ranged from 19 to 94 years, and most participants were middle-aged or elderly. The main characteristics of the included studies are presented in Table 1.

**Table 1:** General characteristics of the included risk models.

Study	Region	Validation population	Study design	Surgery type	Timeline	No. of participants (male/female)	Age (Mean ± SD)	Assessment tool	Diagnostic criteria	Follow-up period
Tang 2025 (23)	Shaoyang, Hunan, China	Shaoyang Central Hospital	Retrospective	MVD	August 2015 to August 2024	187 (82/105)	Relapse group: 57.71 ± 6.04 Nonrecurrence group: 58.58 ± 4.86	NR	NR	24
Xia 2024 (28)	Liaoning, China	Liaoning Provincial People’s Hospital	Prospective	PBC	January 2022 to January 2023	395 (208/187)	66 (range36-89)	Barrow Neurological Institute Pain Score	Postoperative BNI Pain Intensity Grade increased by 1 grade or more compared to the immediate postoperative BNI grade	12
Wang 2024 (29)	Heilongjiang, China	Modeling group: The Fourth Affiliated Hospital of Harbin Medical University; validation group: The First Affiliated Hospital of Harbin Medical University	Retrospective	PBC	January 2020 to December 2023	Modeling group: 317 Validation group: 131	NR	Barrow Neurological Institute Pain Score	Postoperative pain recurrence defined as BNI grade III–V or rehospitalization for surgery during follow-up in patients who initially achieved complete pain relief (BNI grade I)	4–48
Sun 2024 (30)	Qingdao, Shandong, China	Qingdao Haici Hospital Affiliated to Qingdao University	Retrospective	PRT	January 2020 to December 2021	297	Range 19–79 years	NR	Recurrence of paroxysmal pain during 24-month follow-up	24
Qi 2024 (31)	Huaian, Jiangsu, China	The Second People’s Hospital of Huaian	Retrospective	PBC	April 2019 to September 2021	Training set: 167 (78/89) Test set: 94 (42/52)	Training set: 60.5 ± 3.8 Test set: 60.3 ± 3.7	Barrow Neurological Institute Pain Score	Postoperative pain intensity ≥ preoperative intensity. Postoperative analgesic dosage ≥ preoperative dosage. Requirement for additional surgical intervention	35 (training); 33 (test)
Yu 2024 (24)	Jiaxing, Zhejiang, China	Affiliated Hospital of Jiaxing University	Retrospective	PBC/RF	March 2017 to January 2021	131	NR	Numeric Rating Scale	Postoperative NRS-11 score less than 50% of the preoperative score	24
Wu 2024 (25)	Wuhan, China	Department of Neurosurgery, Zhongnan Hospital, Wuhan University	Retrospective	PBC	January 2017 to March 2023	117 (43/74)	Ineffective group: 65.69 ± 10.849 Effective group: 69.69 ± 9.536	Barrow Neurological Institute Pain Score	BNI grade IV-V indicates ineffective pain relief	NR
Peng 2024 (26)	Beijing, China	Beijing Shijitan Hospital, Capital Medical University	Retrospective	MVD	December 2008 to July 2022	124 (56/68)	NR	Barrow Neurological Institute Pain Score	Postoperative BNI I-II transitioning to BNI III-V after ≥1 year, with clinical symptoms meeting TN diagnostic criteria	16.5 ± 2.4
Li 2024 (27)	Shanghai, China; Xiamen, China	A: Shanghai Ninth People’s Hospital, Shanghai Jiao Tong University School of Medicine. B: Zhongshan Hospital of Xiamen University, School of Medicine, Xiamen University. C: The First Affiliated Hospital of Xiamen University	Retrospective	MVD	March 2016 to March 2023	455	NR	Barrow Neurological Institute Pain Score	No significant pain relief upon discharge or pain recurrence during follow-up (BNI ≥ IIIa)	Development cohort: median 34 (range 6–89). Test cohort: median 35 (range 6–91)
Li 2023 (36)	Chengde, Hebei, China	Department of Neurosurgery, Affiliated Hospital of Chengde Medical College	Retrospective	PBC	November 2018 to December 2021	60 (17/43)	63.90 ± 10.76	Barrow Neurological Institute Pain Score	BNI pain score grade III–V at 12 months postoperatively	12
Wu 2023 (32)	Zhejiang, China	Affiliated Hospital of Jiaxing University	Retrospective	RF	April 2014 to December 2020	196	NR	Numeric Rating Scale	(Preoperative NRS-11 – Postoperative NRS-11)/Preoperative NRS-11 < 50%	24
Wang 2023 (33)	Hefei, Anhui, China	The Second Affiliated Hospital of Anhui Medical University	Retrospective	MVD	August 2011 to October 2021	56 (23/33)	59 (range 25–77)	Barrow Neurological Institute Pain Score	BNI pain intensity score II-V	12
Deng 2023 (34)	Jiaxing, Zhejiang, China	Affiliated Hospital of Jiaxing University	Retrospective	RF	February 2016 to August 2019	139	NR	Numeric Rating Scale	The reappearance of pain symptoms during follow-up, with cumulative survival rates analyzed by Kaplan–Meier method	24
Chen 2023 (35)	Zhejiang, China	Affiliated Hospital of Jiaxing University	Retrospective	PBC/RF	January 2016 to April 2021	155	NR	Numeric Rating Scale	(Preoperative NRS-11 – Postoperative NRS-11)/Preoperative NRS-11 < 50%	12
Zhao 2022 (38)	Zhejiang, China	People’s Hospital of Rudong (Nantong) and Affiliated Hospital of Jiaxing University	Retrospective	PBC/PRT	February 2016 to September 2020	123 (50/73)	34–94	Numeric Rating Scale	NRS reduction <50% compared to preoperative baseline	12
Pang 2022 (39)	Guangxi, China	Second Affiliated Hospital of Guangxi Medical University	Retrospective	PBC	May 2019 to December 2020	134 (62/72)	NR	Barrow Neurological Institute Pain Score	Patients with initial BNI grade I or II pain progressing to BNI grade III, IV, or V during follow-up	12
Kourilsky 2022 (37)	France and USA	Hospital Foundation Adolphe de Rothschild (Paris) and Hospital Henri Mondor (Paris)	Retrospective	PBC	1985 to 2019	131 (45/86)	75	Clinical evaluation by neurosurgeon	Recurrence of preoperative shooting pain + ≥ 1 of: 1. Pain intensity ≥ preoperative level (surgeon’s evaluation); 2. Need for pain medications at doses ≥ preoperative or additional drugs; 3. Requirement of a second surgical procedure	36
Zhang 2021 (40)	Changsha, Hunan, China	Department of Neurosurgery, the First Affiliated Hospital of Hunan University of Chinese Medicine	Prospective	MVD	June 2016 to December 2018	260	Modeling group: 56.70 ± 11.45. Validation group: 55.82 ± 10.97	Clinical follow-up records and medication status	Recurrent paroxysmal electric shock-like or needle-like severe pain requiring high-dose carbamazepine treatment	12
Li 2021 (41)	Lianyungang, Jiangsu, China	Lianyungang Hospital, Xuzhou Medical University	Retrospective	PRT	June 2013 to December 2019	438 (202/266)	58.69 ± 6.12	Barrow Neurological Institute Pain Score	Reappearance of trigeminal neuralgia symptoms more than 3 months after surgery	Median 49
Shi 2020 (42)	Changzhou, China	Third Affiliated Hospital of Soochow University	Retrospective	MVD	March 2008 to March 2016	184	NR	Barrow Neurological Institute Pain Score	BNI scores of III–V	3–48

MVD, microvascular decompression; PBC, percutaneous balloon compression; PRT, percutaneous trigeminal radiofrequency thermocoagulation; RF, percutaneous foramen ovale radiofrequency; BNI, barrow neurological institute; NRS, Numeric Rating Scale.

The included studies varied substantially in follow-up duration. These ranged from 3 months to 91 months. Follow-up assessments were frequently performed at 12 and 24 months across multiple studies. Definitions of recurrence also differed considerably among the studies. Commonly adopted definitions included an increase in Barrow Neurological Institute (BNI) pain score, repeat hospitalization for surgical intervention, return of pain to preoperative levels, or requirement for additional treatment. Recurrence was most commonly assessed using the BNI pain score, the 11-point Numerical Rating Scale (NRS-11), symptom recurrence, and changes in medication requirements.

In summary, studies investigating the prediction of pain recurrence following trigeminal neuralgia surgery vary considerably in surgical procedures, patient cohorts, follow-up durations, and definitions of recurrence. Nevertheless, these studies provide valuable data that can inform the development of predictive models.

### 3.3 Characteristics of prediction models

Details of the prediction models are provided in Supplementary material 2. These models employed a variety of approaches, encompassing both conventional statistical methods and machine learning techniques. Fifteen studies (23, 24, 26, 29–33, 35, 36, 38–42) utilized logistic regression as the primary modeling approach. Four studies (27, 28, 34, 37) applied Cox regression for survival analysis. Two studies (25, 29) employed machine learning algorithms, specifically random forest and XGBoost. The prediction models were presented in various formats, most commonly nomograms, mathematical formulas, or online dynamic calculators, with nomograms being the most frequently adopted owing to their intuitive visualization and clinical utility. Model validation approaches varied considerably, ranging from internal validation only to external validation; nonetheless, external validation remained uncommon and was reported in only a limited number of studies (27, 29). Most models exhibited satisfactory discriminative ability, with area under the curve (AUC) values predominantly ranging from 0.70 to 0.90; several models even achieved AUCs exceeding 0.90, indicating strong performance in identifying recurrence risk. Calibration was primarily evaluated using calibration plots or the Hosmer–Lemeshow test, with the majority of models demonstrating acceptable performance, although some exhibited predictive bias at extreme probability values. Decision curve analysis (DCA) indicated that a substantial proportion of models yielded positive net benefit across clinically relevant threshold ranges, supporting their potential clinical utility.

### 3.4 Quality assessment

As shown in Table 2, the PROBAST assessment indicated that one model (28) had a low risk of bias, one model (40) had an unclear risk of bias, and the remaining 18 models (23–27, 29–39, 41, 42) exhibited a high risk of bias. In the participant domain, 18 retrospective studies (23–27, 29–39, 41, 42) were judged to have a high risk of bias owing to their retrospective design, whereas the two prospective studies (28, 40) were rated as having a low risk of bias. In the predictors domain, 14 retrospective studies (23–25, 27, 29–31, 33, 34, 36–39, 41) were assigned an unclear risk of bias because measures to minimize bias (e.g., blinding) were not reported, whereas although retrospective in design, four studies (26, 32, 35, 42) were rated as having a low risk of bias owing to the implementation of blinding procedures. The two prospective studies (28, 40) were rated as having a low risk of bias. In the outcome domain, two studies (23, 29) were assigned an unclear risk of bias owing to the absence of a standardized outcome definition and the use of vague descriptions, whereas the remaining 19 studies (24–28, 30–42) were judged to have a low risk of bias. In the analysis domain, 13 studies (23–26, 30, 34–40, 42) were assigned an unclear risk of bias, whereas seven studies (27–29, 31–33, 41) were judged to have a low risk of bias. With respect to sample size, all 20 studies (23–42) were rated as having a low risk of bias, as the sample sizes were adequate or the events per variable (EPV) exceeded 20. In the data handling domain, one study (40) received an unclear risk of bias owing to the lack of reported methods for handling missing data. Regarding model performance evaluation, 10 studies (23, 27–33, 36, 41) performed a comprehensive assessment, whereas 10 studies (24–26, 34, 35, 37–40, 42) failed to report the evaluation methods employed. In the model validation domain, 16 studies (24–29, 31–35, 37–41) conducted internal or external validation and were therefore rated as having a low risk of bias, whereas four studies (23, 30, 36, 42) received an unclear risk owing to unreported validation procedures. Because only one model was judged to be at low risk of bias, stratified meta-analysis according to risk-of-bias level was not feasible, which further limits confidence in the pooled estimates.

**Table 2:** Evaluations of the bias risk and applicability of the included studies.

References	Risk of bias				Risk of applicability			Overall risk	
	Participants	Predictors	Outcome	Analysis	Participants	Predictors	Outcome	Risk of bias	Risk of applicability
Tang 2025 (23)	High	Unclear	Unclear	Unclear	Low	Low	Low	High	Low
Xia 2024 (28)	Low	Low	Low	Low	Low	Low	Low	Low	Low
Wang 2024 (29)	High	Unclear	Unclear	Low	Low	Low	Low	High	Low
Sun 2024 (30)	High	Unclear	Low	Unclear	Low	Low	Low	High	Low
Qi 2024 (31)	High	Unclear	Low	Low	Low	Low	Low	High	Low
Yu 2024 (24)	High	Unclear	Low	Unclear	Low	Low	Low	High	Low
Wu 2024 (25)	High	Unclear	Low	Unclear	Low	Unclear	Unclear	High	Unclear
Peng 2024 (26)	High	Low	Low	Unclear	Low	Low	Low	High	Low
Li 2024 (27)	High	Unclear	Low	Low	Low	Low	Low	High	Low
Li 2023 (36)	High	Unclear	Low	Unclear	Low	Low	Low	High	Low
Wu 2023 (32)	High	Low	Low	Low	Low	Low	Low	High	Low
Wang 2023 (33)	High	Unclear	Low	Low	Low	Low	Low	High	Low
Deng 2023 (34)	High	Unclear	Low	Unclear	Low	Low	Low	High	Low
Chen 2023 (35)	High	Low	Low	Unclear	Low	Low	Low	High	Low
Zhao 2022 (38)	High	Unclear	Low	Unclear	Low	Low	Low	High	Low
Pang 2022 (39)	High	Unclear	Low	Unclear	Low	Low	Low	High	Low
Kourilsky 2022 (37)	High	Unclear	Low	Unclear	Low	Low	Low	High	Low
Zhang 2021 (40)	Low	Low	Low	Unclear	Low	Low	Low	Unclear	Low
Li 2021 (41)	High	Unclear	Low	Low	Low	Low	Low	High	Low
Shi 2020 (42)	High	Low	Low	Unclear	Low	Low	Low	High	Low

High, high risk of bias or high applicability concern; Low, low risk of bias or low applicability concern; Unclear, unclear risk of bias or unclear applicability concern.

The applicability risks are summarized in Table 2. Because the follow-up duration was not reported for Wu’s model (25), the applicability risk remains unclear in the “Predictors” and “Outcome” domains. Ultimately, 19 models (23, 24, 26–42) were judged to have low overall applicability concerns, while one model (25) was judged to have unclear overall applicability concerns.

## 4 Meta-analysis of C-statistic

### 4.1 Results of the training set models

**Figure 2:**
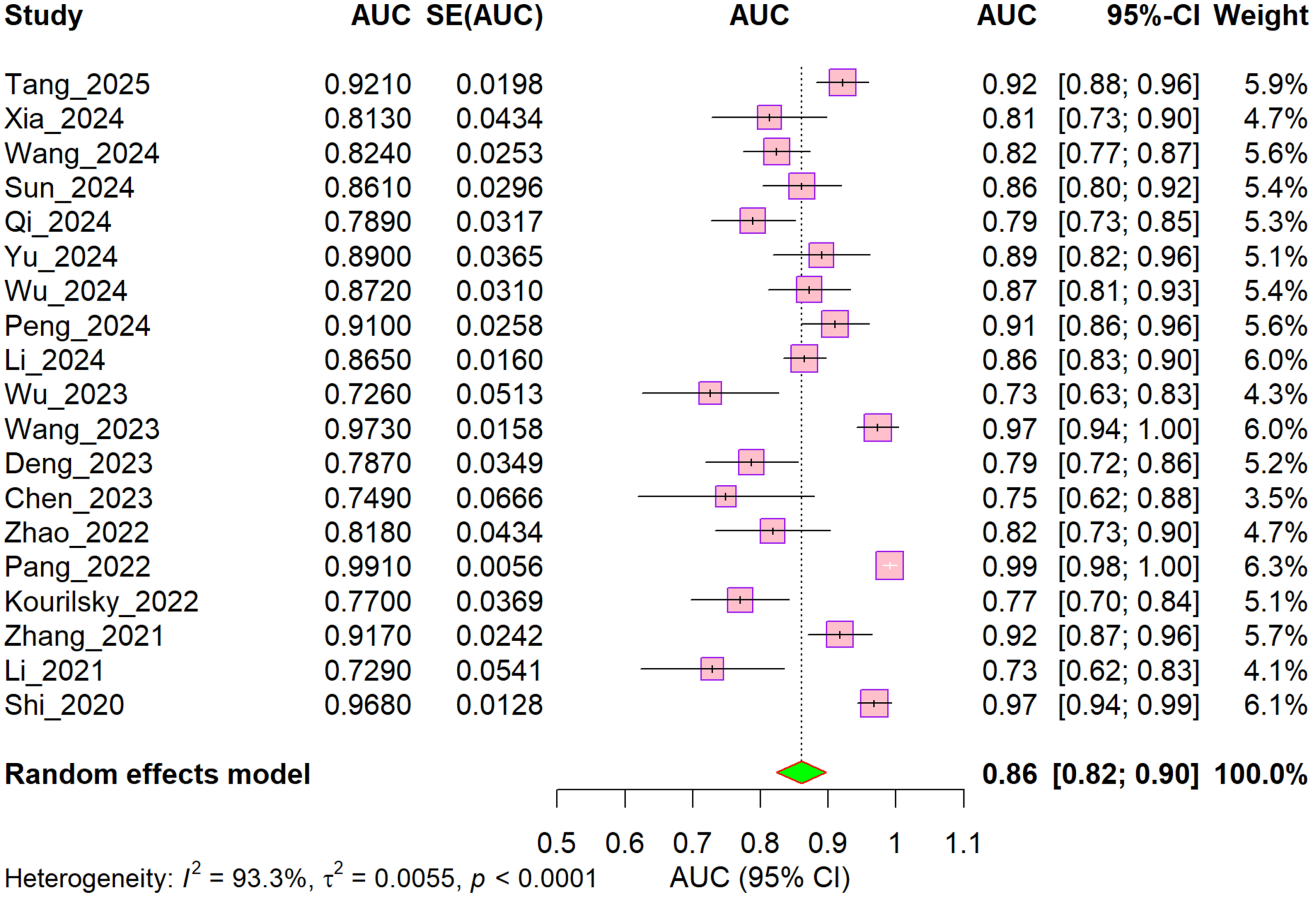
Forest plot of the training set models.

Figure 2 presents the AUC values of the prediction models in the training sets across the included studies, along with the results of the heterogeneity analysis. The AUC values reported in the 19 included studies (23–41) ranged from 0.726 (32) to 0.991 (39); one study (42) was excluded because it reported only the AUC for individual predictive factors rather than the overall model. The random-effects pooled estimate of the AUC in the training sets was 0.86 (95% CI: 0.82–0.90), suggesting good overall discriminative performance across studies. Nevertheless, substantial heterogeneity was observed among the studies (I^2^ = 93.3%, τ^2^ = 0.0055, *p* < 0.001), indicating considerable variation in predictive performance across studies.

### 4.2 Publication bias assessment

**Figure 3:**
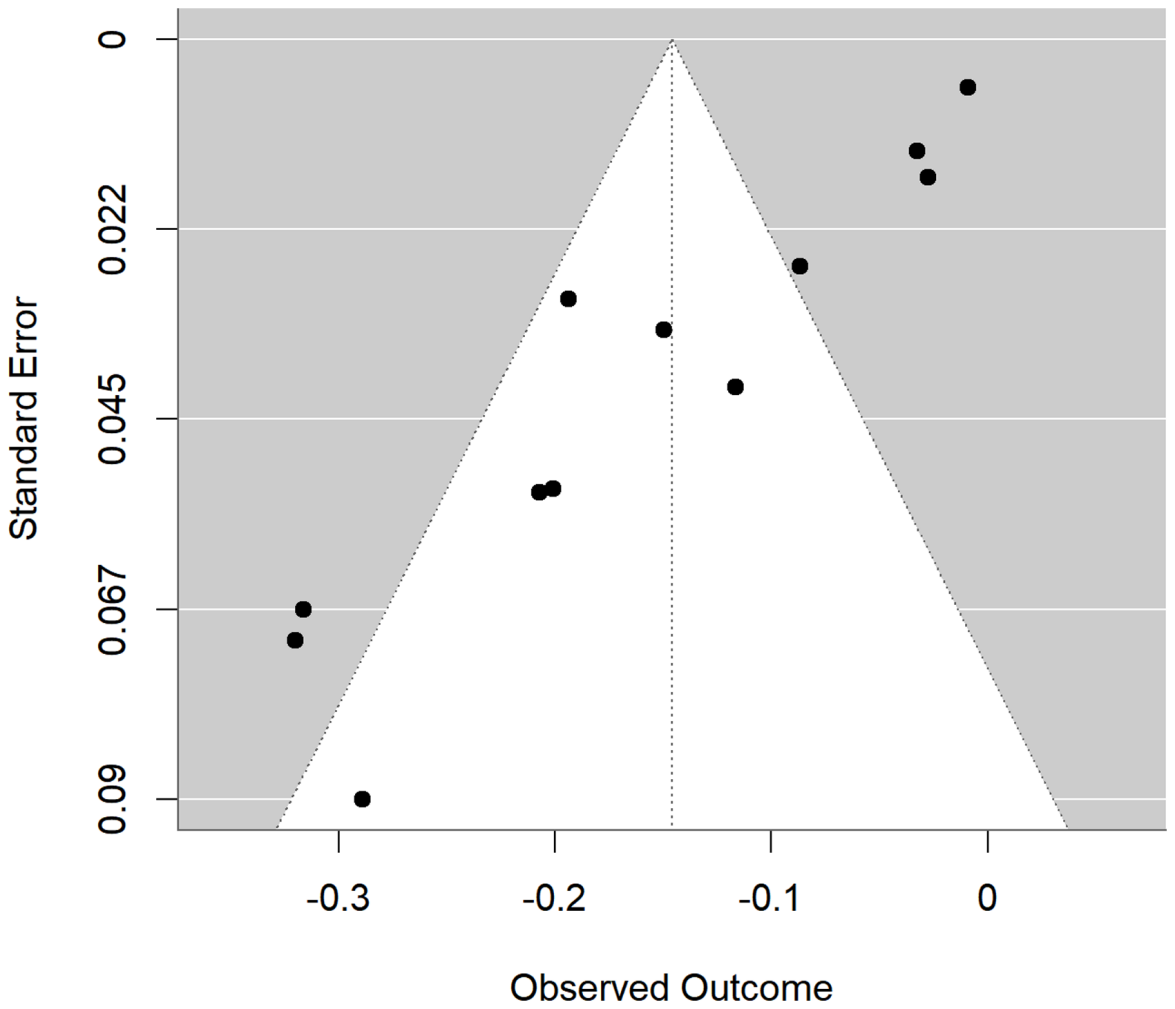
Funnel plot of training set models.

Figure 3 shows that the funnel plot of AUC values in the training set exhibits a marked concentration of points on the right side (higher AUC values) with sparse and asymmetrically distributed points on the left side (lower AUC values), suggesting the likely presence of publication bias or a small-study effect. This asymmetry is corroborated by Egger’s test (t = −9.5887, df = 10, *p* < 0.001), which yielded a highly significant result (*p* < 0.001), confirming the presence of statistically significant publication bias. Consequently, the AUC analysis in the training set is at substantial risk of publication bias, underscoring the need for sensitivity analyses to assess the robustness of these results.

### 4.3 Results of the validation set models

**Figure 4:**
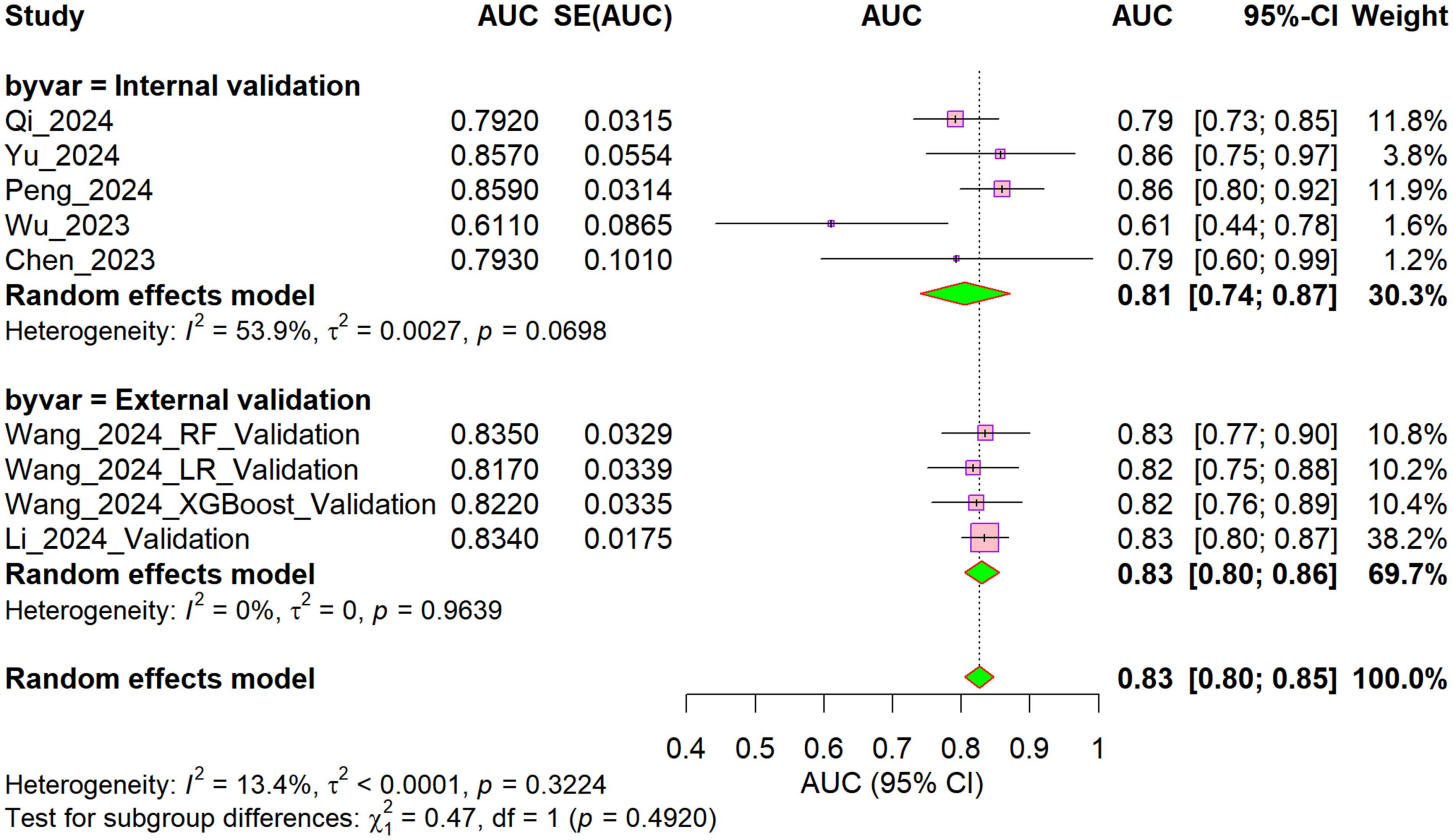
Forest plot of the validation set models.

Five studies (24, 26, 31, 32, 35) were included in the internal validation set. The AUC values across individual studies ranged from 0.611 to 0.859, with a pooled AUC of 0.81 (95% CI: 0.74–0.87), albeit with substantial heterogeneity (I^2^ = 53.9%). In the external validation set, comprising two studies (27, 29) (four models), AUC values ranged from 0.817 to 0.835, suggesting more consistent performance. The pooled AUC was 0.83 (95% CI: 0.80–0.86), with no detectable heterogeneity (I^2^ = 0%). Combining internal and external validation results yielded a pooled AUC of 0.83 (95% CI: 0.80–0.85) (Figure 4). Subgroup analysis revealed no significant difference in AUC between the internal and external validation sets (χ^2^ = 0.47, *p* = 0.492), with low heterogeneity (I^2^ = 13.4%). However, limited evidence suggests consistent performance, but external validation is scarce and geographically restricted.

### 4.4 Subgroup analysis

#### 4.4.1 Type of surgery

**Figure 5:**
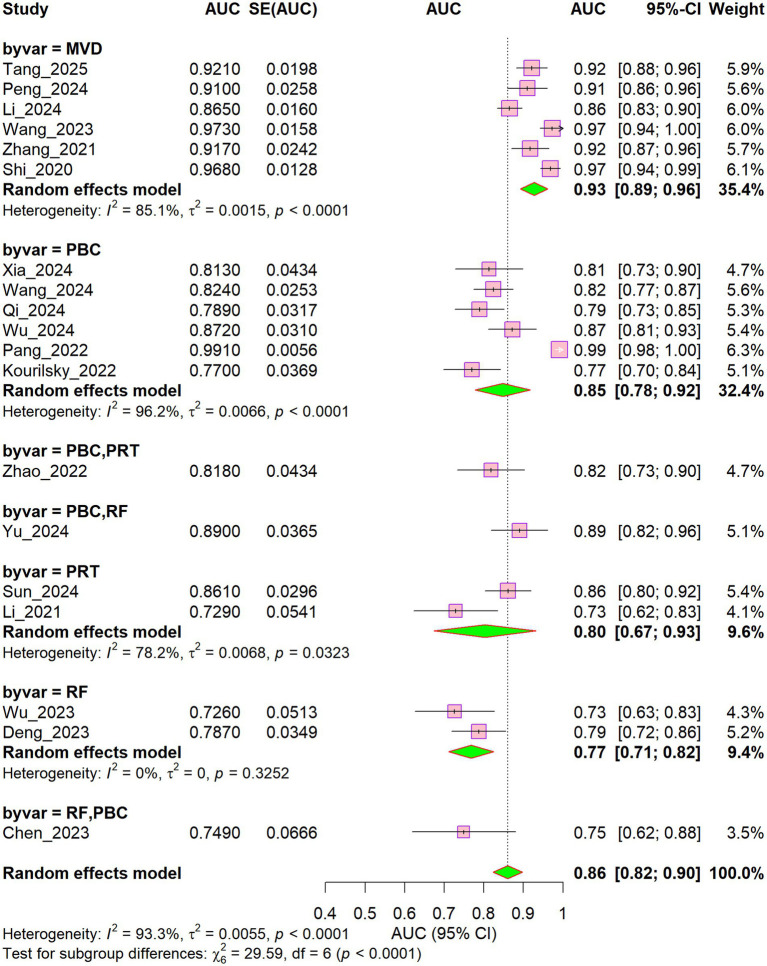
Subgroup forest plot of different surgical types.

Substantial differences in the area under the curve (AUC) of the predictive models were observed across surgical types (Figure 5). The MVD subgroup model demonstrated the highest performance, yielding a pooled AUC of 0.93 (95% CI: 0.89–0.96; I^2^ = 85.1%) and contributing a weight of 35.4%. For the remaining surgical types (e.g., PBC, PRT, and RF), AUC values ranged predominantly from 0.77 to 0.86, accompanied by generally high heterogeneity. The overall difference between subgroups was statistically significant (χ^2^ = 29.59, df = 6, *p* < 0.001), indicating that surgical type exerts a substantial influence on model predictive performance. Accordingly, appropriate model selection based on the specific surgical procedure is recommended for clinical applications.

#### 4.4.2 Pain rating scale

**Figure 6:**
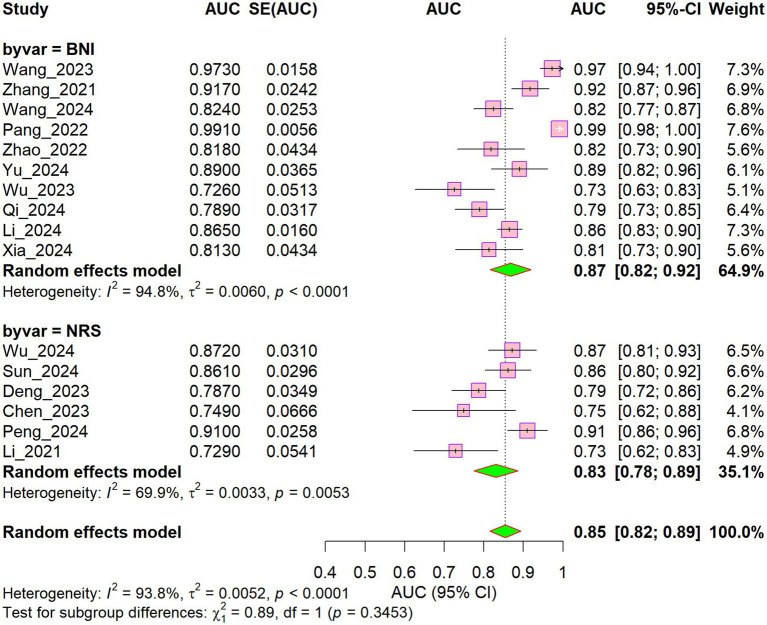
Subgroup forest plot of different scales.

Stratified by pain assessment scale (Figure 6), the included studies were categorized into two subgroups: the BNI subgroup (9 studies) yielded an AUC of 0.87 (95% CI: 0.82–0.92; I^2^ = 94.8%) with a weight of 64.9%, while the NRS subgroup (5 studies) yielded an AUC of 0.83 (95% CI: 0.78–0.89; I^2^ = 69.9%) with a weight of 35.1%. The overall pooled AUC was 0.85 (95% CI: 0.82–0.89; I^2^ = 93.8%). The difference in AUC between the two subgroups was not statistically significant (*p* = 0.345). Nevertheless, the BNI scale showed a numerically superior discriminatory performance compared with the NRS scale.

#### 4.4.3 Development methodology

**Figure 7:**
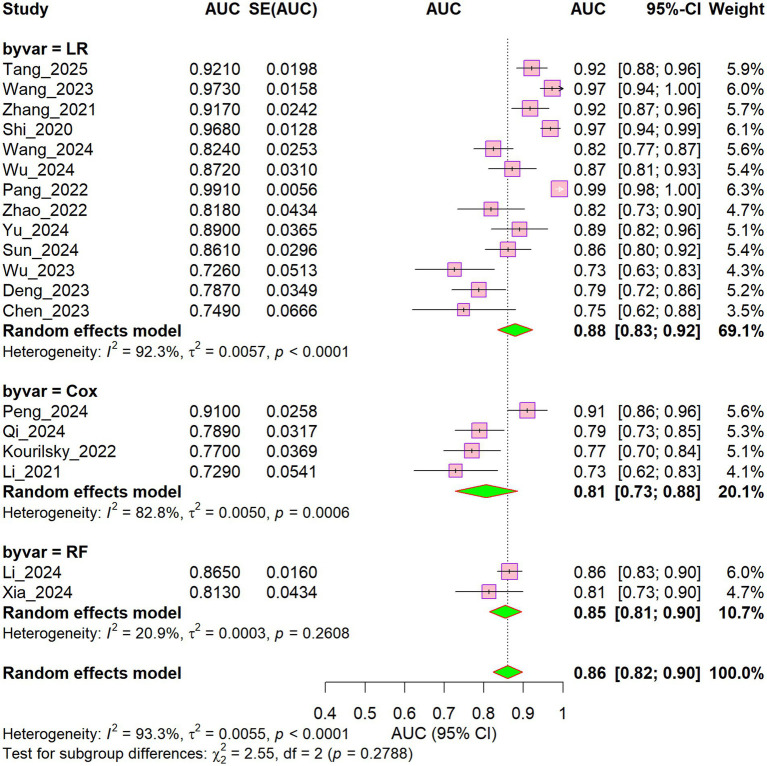
Subgroup forest plot of development methodology.

The pooled areas under the curve (AUCs) were 0.88 (I^2^ = 92.3%) for the logistic regression (LR) model, 0.81 (I^2^ = 82.8%) for the Cox regression model, and 0.85 (I^2^ = 20.9%) for the random forest (RF) model (Figure 7). Although the LR model achieved the highest pooled AUC, it also displayed the greatest heterogeneity. In contrast, the RF model showed the lowest heterogeneity (I^2^ = 20.9%), indicating that the choice of modeling methodology may substantially affect model stability.

In summary, surgical procedure, outcome measures, and modeling approach each exerted a significant influence on model discrimination (with generally high heterogeneity observed across subgroups). Subgroup analyses revealed statistically significant between-group differences for surgical procedure and modeling approach (both *p* < 0.001).

### 4.5 Sensitivity analysis

**Figure 8:**
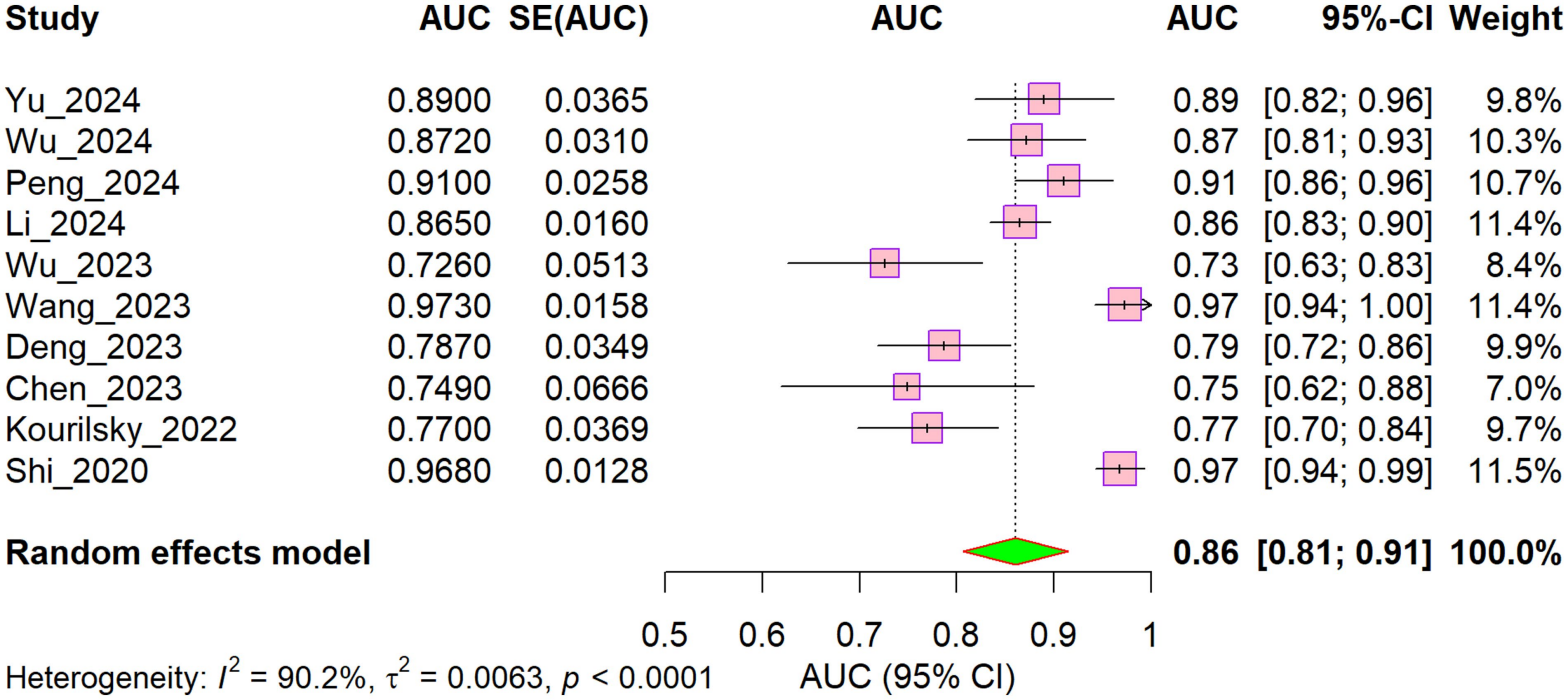
Forest map included in English literature.

To evaluate the robustness of the pooled discriminative performance, we conducted a sensitivity analysis by excluding Chinese-language studies and including only English-language publications (Figure 8; Table 3). Following this restriction, the pooled AUC remained virtually unchanged at 0.86 (95% CI: 0.81–0.91) compared with 0.86 (95% CI: 0.82–0.90) in the primary analysis. Although between-study heterogeneity decreased modestly (I^2^ decreased from 93.3 to 90.2%), it remained high. These results suggest that the overall predictive performance is stable and not substantially influenced by the inclusion of Chinese-language studies, thereby supporting the robustness of the main findings across language subgroups.

**Table 3:** Comparison of sensitivity analysis results based on full sample and English only sample.

Analysis scenario	Number of included studies	Pooled AUC (95% CI)	I^2^	Interpretation
Overall analysis	19	0.86 (0.82–0.90)	93.30%	Prediction models demonstrated good pooled discrimination in the overall dataset.
	(9 Chinese-language, 10 English-language)			
Sensitivity analysis excluding Chinese-language studies	10	0.86 (0.81–0.91)	90.20%	Exclusion of Chinese-language studies did not materially alter the pooled estimate, supporting the robustness of the primary result. Residual heterogeneity remained high.

**Figure 9:**
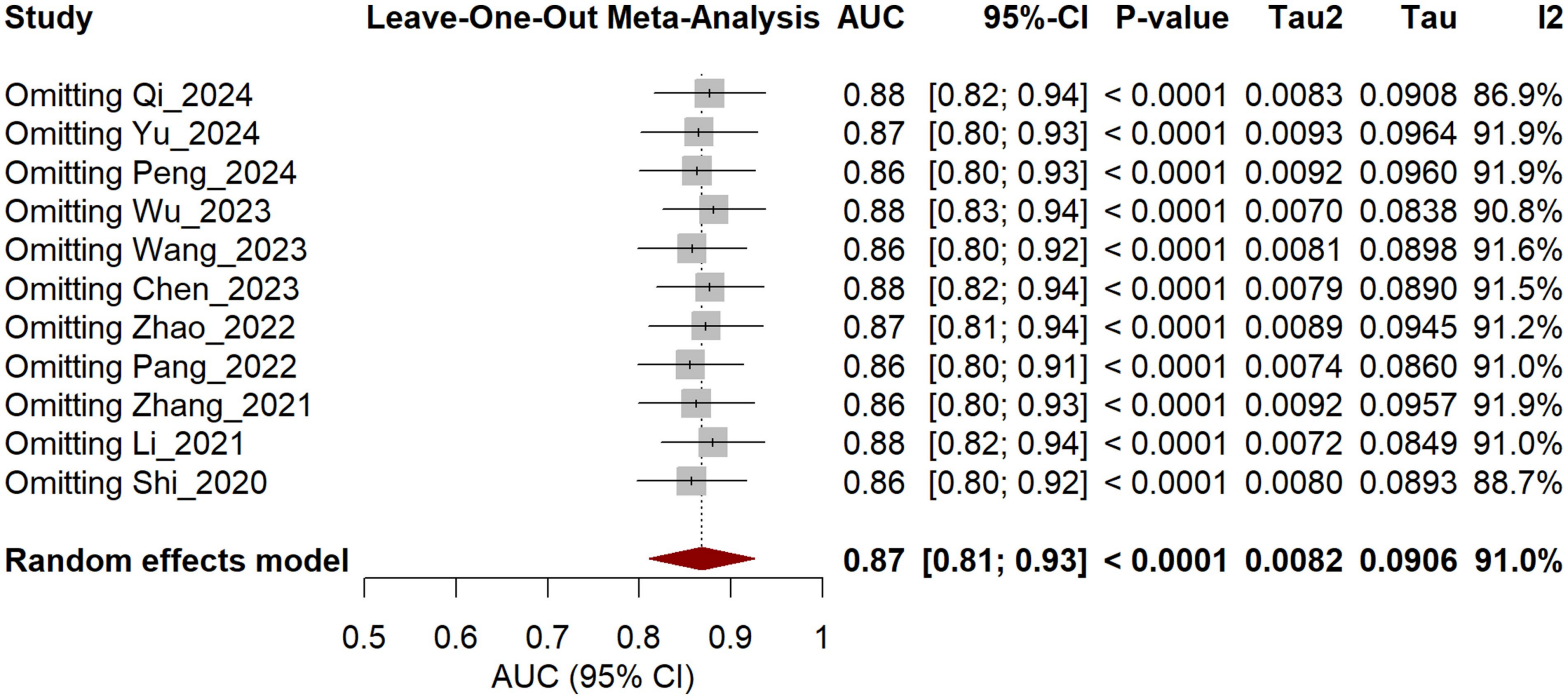
Sensitivity analysis of training set.

**Figure 10:**
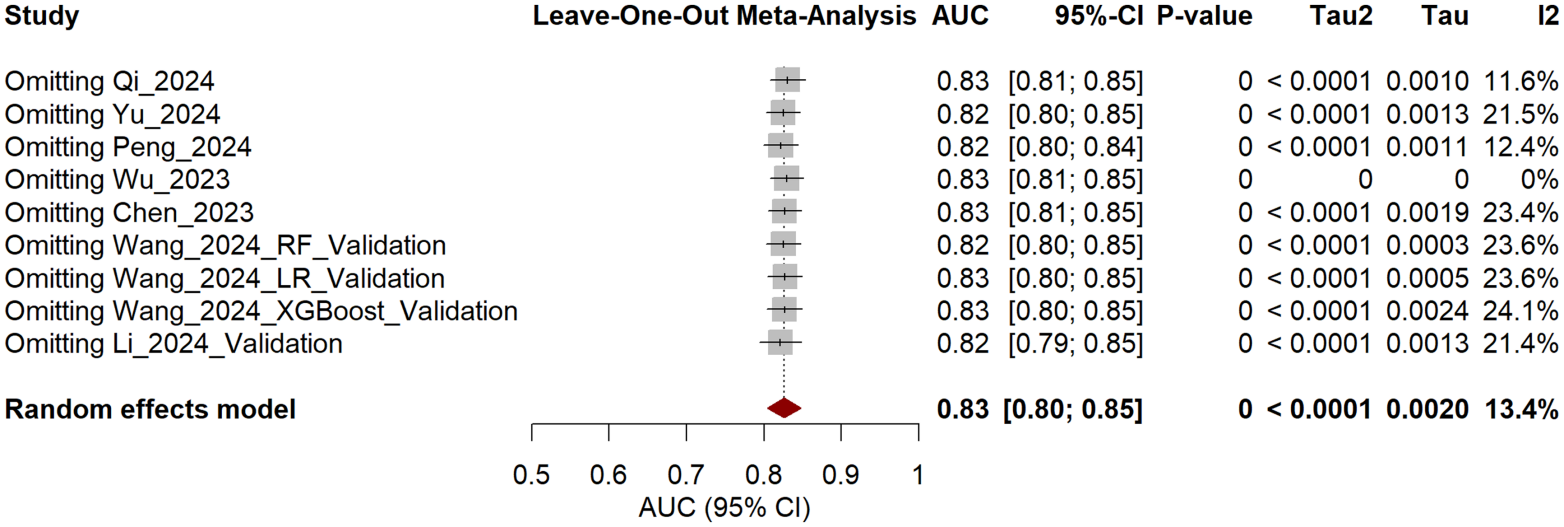
Sensitivity analysis of validation set.

To evaluate the influence of individual studies on the pooled results, a leave-one-out sensitivity analysis was conducted. The developed model for predicting postoperative recurrence of trigeminal neuralgia demonstrated strong predictive performance. In the training cohort, the area under the curve (AUC) ranged from 0.86 to 0.88 (Figure 9). In the validation cohort, the AUC ranged from 0.82 to 0.83 (Figure 10). All obtained AUC values achieved statistical significance (all *p* < 0.001). The model exhibited robust stability, with no single study exerting a substantial influence on the overall findings. These results indicate that the model is reliable and holds potential clinical utility.

### 4.6 Predictors of risk prediction models

**Figure 11:**
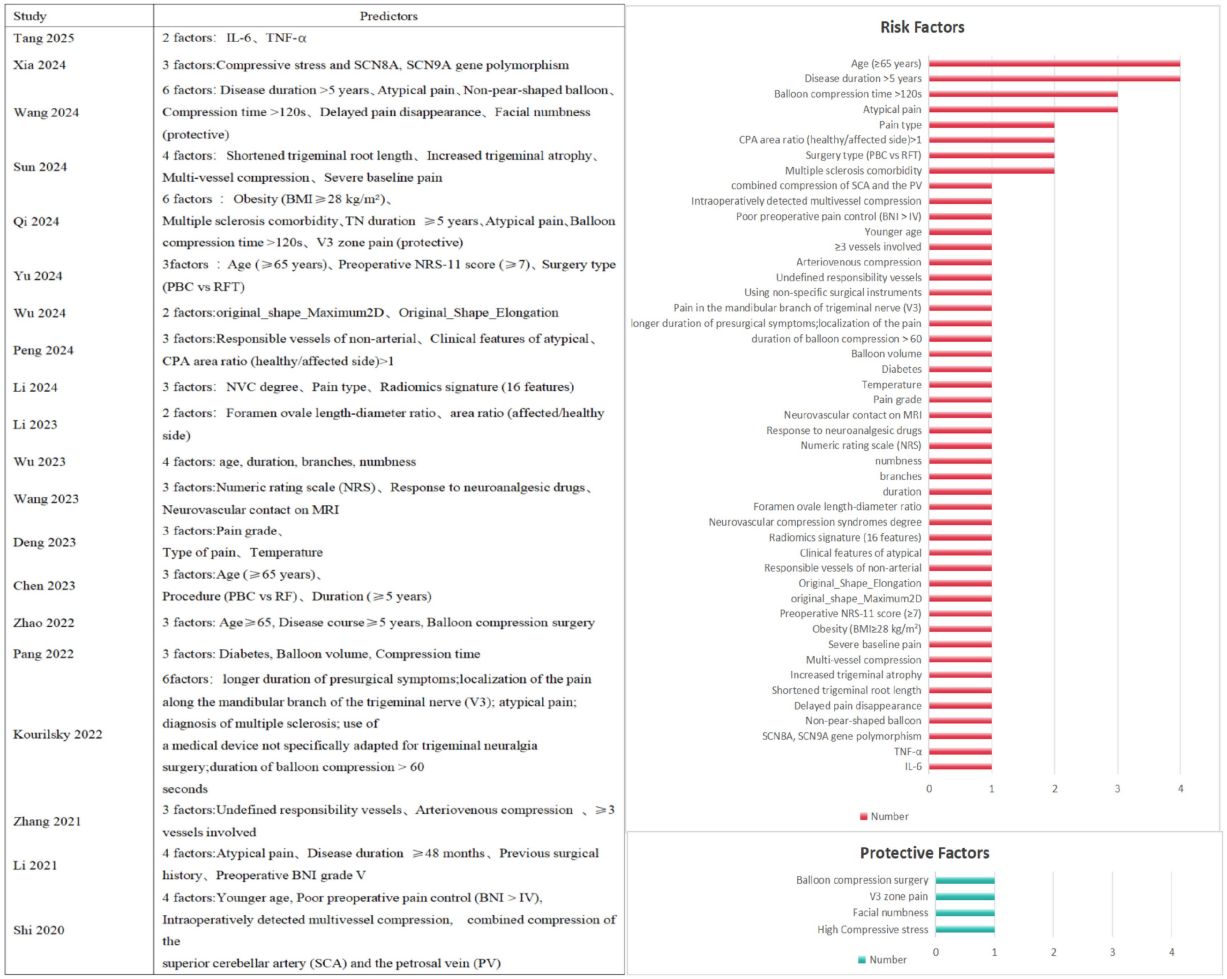
Prediction factor frequency chart.

The models incorporated a total of 51 predictors. Of these, 47 were risk factors and four were protective factors (Figure 11). Certain predictors appeared more frequently across the models, occurring in two to four of them. The most frequently included predictors were: disease duration >5 years, age ≥65 years, atypical pain, balloon compression time >120 s, multiple sclerosis, surgical procedure type, CPA area ratio (healthy/affected side) > 1, and pain type.

**Figure 12:**
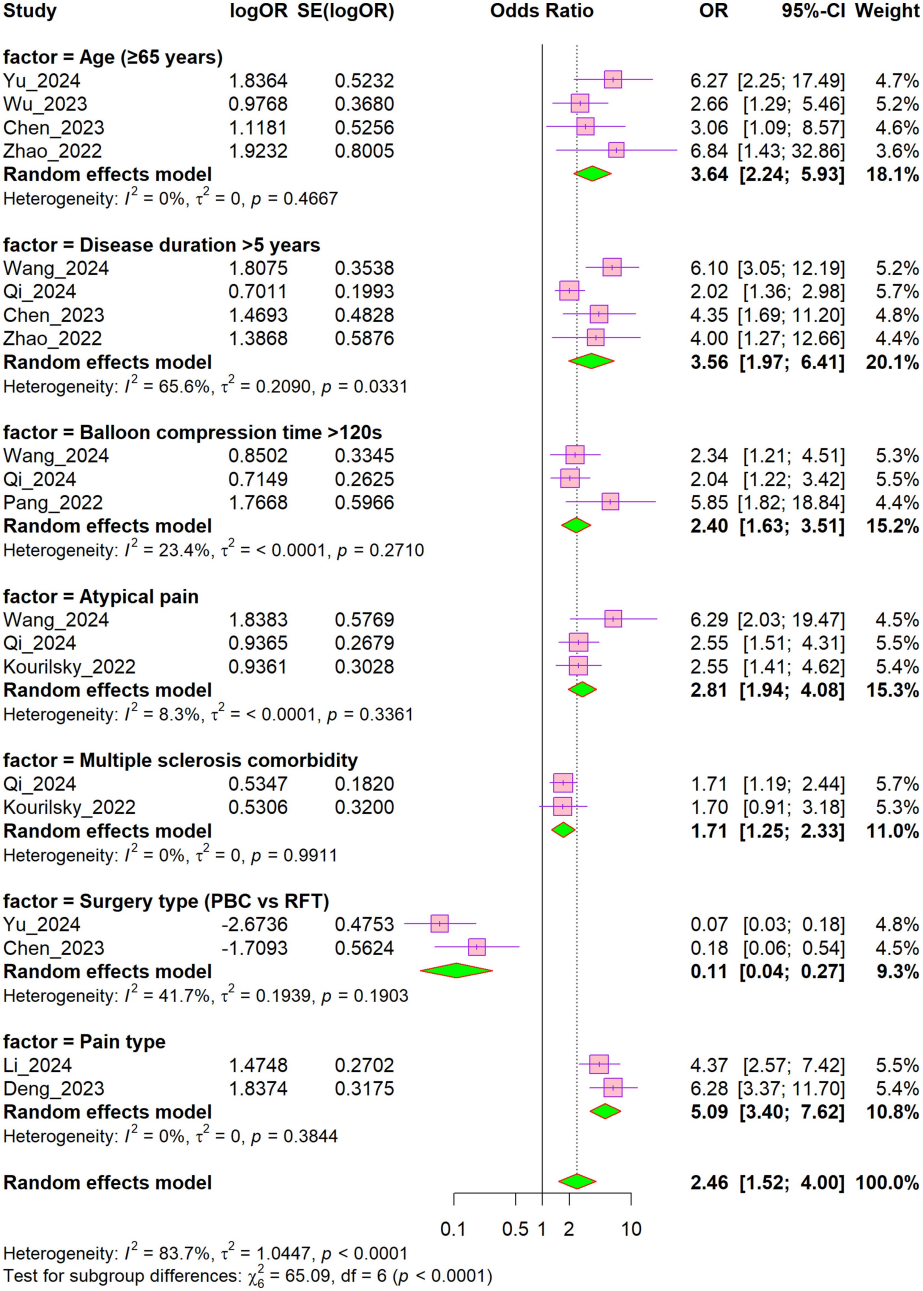
Forest plot of odds ratios for high frequency predictors.

This meta-analysis synthesized data from multiple risk prediction models to identify factors associated with recurrence of trigeminal neuralgia following surgery (Figure 12). Age emerged as a prominent predictor: patients aged 65 years or older exhibited an approximately fourfold increased risk of recurrence compared with younger individuals (pooled OR = 3.64). Likewise, a disease duration exceeding 5 years was associated with a substantially elevated risk of recurrence (pooled OR = 3.56), albeit with considerable heterogeneity among the included studies (I^2^ = 65.6%). Prolonged balloon compression (>120 s) during surgery was also associated with an increased risk of recurrence (pooled OR = 2.40), with low heterogeneity across studies (I^2^ = 23.4%). Atypical pain symptoms were similarly associated with an elevated recurrence risk (pooled OR = 2.81). In contrast, the surgical modality significantly influenced recurrence risk: percutaneous balloon compression (PBC) was associated with a markedly lower risk compared with radiofrequency thermocoagulation (RFT) (pooled OR = 0.11). Pain type demonstrated a strong association with recurrence (pooled OR = 5.09), with no heterogeneity across studies (I^2^ = 0%), indicating high reliability as a predictor. Although multiple sclerosis (MS) was not a statistically significant predictor of recurrence (pooled OR = 1.71, *p* > 0.05), the effect size suggests that MS may confer a clinically relevant increased risk. The lack of statistical significance is likely attributable to the limited number of TN patients with comorbid MS, resulting in insufficient statistical power. Of note, the results for MS exhibited no heterogeneity across studies (I^2^ = 0%). In summary, age, disease duration, surgical modality, and pain type represent the principal factors to consider when assessing an individual patient’s risk of trigeminal neuralgia recurrence.

## 5 Discussion

### 5.1 Key findings

This is the first study to systematically review and meta-analyze prediction models for recurrence of trigeminal neuralgia (TN) following surgical intervention. Across 20 included studies, the existing prediction models showed pooled AUC estimates of 0.86 in development cohorts and 0.83 in validation cohorts, but these summary values likely overestimate true predictive performance because most models were at high risk of bias, publication bias was evident, and heterogeneity was substantial. Therefore, they should not be interpreted as indicating clinically reliable models. Model performance was also observed to vary substantially depending on the specific surgical procedure employed and the methodological framework used for model construction. Moreover, only two studies performed external validation, and most evidence came from Chinese cohorts, limiting the generalizability of the findings. These findings have significant implications for clinicians utilizing such predictive tools in clinical decision-making and for researchers developing future TN recurrence prediction models.

Furthermore, we identified several highly consistent predictors, including advanced age (≥65 years), prolonged disease duration (>5 years), atypical pain characteristics, and certain surgical procedures. These factors provide an important foundation for individualized risk assessment.

### 5.2 In depth exploration of model performance

This meta-analysis demonstrated that the prediction models exhibited strong discriminatory performance, despite considerable heterogeneity among the included studies. The pooled AUC in the validation set was 0.83, with low between-study heterogeneity (I^2^ = 13.4%). This performance surpasses that reported in most individual studies. These findings indicate that pooling data across multiple studies yields more reliable estimates of model performance (19). Subgroup analysis by patient population yielded clear findings, revealing that the type of surgery significantly influenced model predictive performance. The model incorporating MVD data demonstrated the highest performance (AUC = 0.93) and was most accurate in identifying patients likely to achieve favorable outcomes. This superior performance is likely attributable to the fact that microvascular decompression (MVD) directly targets the primary etiology of trigeminal neuralgia (TN)—namely, vascular compression of the nerve. Consequently, factors associated with pain recurrence are more closely linked to intraoperative findings during MVD (43). Nevertheless, the exceptionally high AUC in the MVD subgroup should be interpreted cautiously, given the substantial heterogeneity (I^2^ = 85.1%) and the potential for publication bias, which may have led to overestimation of model performance. In contrast, models based on ablative procedures (e.g., percutaneous balloon compression [PBC] or radiofrequency thermocoagulation [RF]) showed greater variability in performance (AUC range 0.77–0.86). This variability likely reflects the complex and multifactorial mechanisms underlying recurrence after neuroablative procedures, including differences in nerve regeneration and lesion extent (44). Subgroup analysis by modeling approach revealed that logistic regression yielded the highest pooled AUC (0.88), albeit with the greatest heterogeneity (I^2^ = 92.3%). Conversely, random forest–based models exhibited substantially lower heterogeneity (I^2^ = 20.9%), suggesting greater stability. These findings suggest that greater incorporation of machine learning algorithms in future model development may enhance both predictive accuracy and robustness. Subgroup analysis stratified by pain assessment scale showed no significant difference in discriminatory ability between models based on the Barrow Neurological Institute (BNI) score and the Numeric Rating Scale (NRS) (*p* = 0.3453), although the BNI subgroup had a slightly higher pooled AUC (0.87 vs. 0.83). This small advantage may stem from the BNI score being specifically designed for trigeminal neuralgia, as it incorporates both pain intensity and medication usage, providing a more comprehensive assessment than the NRS, which measures pain intensity alone. Nevertheless, both subgroups exhibited substantial heterogeneity (I^2^ > 60%), indicating that the choice of scale alone does not account for all performance variation. Other factors, such as study design and patient characteristics, likely contribute more substantially. Accordingly, future studies should prioritize the development and validation of models in well-defined subpopulations, incorporating more robust machine learning techniques and standardized, disease-specific outcome measures to minimize heterogeneity and improve the clinical utility and reliability of these predictive tools.

### 5.3 Pathophysiological mechanisms of key predictive factors

Age ≥65 years emerged as a strong risk factor for recurrence (OR = 3.64), potentially attributable to diminished neuronal repair capacity, heightened central sensitization that is more resistant to reversal, and a greater burden of comorbidities among elderly patients (45). Notably, the predictive effect of advanced age was predominantly observed in patients undergoing microvascular decompression (MVD), whereas it was not significant in those treated with ablative procedures. These findings indicate that the effect of age on recurrence risk varies according to the surgical approach and the specific patient cohort. A prolonged disease duration (>5 years) typically indicates pain chronification, which can induce substantial functional reorganization and structural alterations in the central nervous system, thereby sustaining active pain circuits even after elimination of the initial peripheral trigger; this is consistent with the central sensitization theory underlying the chronicity of trigeminal neuralgia (TN) (7). Atypical pain phenotypes (e.g., persistent burning sensation or hypoesthesia) usually indicate more extensive nerve damage or central mechanistic involvement, and are associated with a higher recurrence risk compared to typical paroxysmal pain; this observation aligns with the more intricate pathophysiological mechanisms encompassed by the concept of “secondary trigeminal neuralgia” as proposed by Lambru et al. (3). As a predictor, surgical type essentially reflects the degree to which various interventions alter the underlying neuropathology. First, this finding corroborates the prevailing consensus that MVD, as an etiology-targeted treatment, theoretically affords superior long-term outcomes relative to symptomatic neurodestructive procedures. Second, our analysis refines the understanding of specific procedures. We observed that percutaneous balloon compression (PBC) exerted a protective effect against recurrence compared with radiofrequency thermocoagulation (RFT), with an odds ratio of 0.11, suggesting a substantially lower recurrence risk with PBC among comparable interventions. Nevertheless, this result warrants cautious interpretation owing to heterogeneous findings in the literature comparing PBC and RFT. Recent evidence supports a lower medium-term recurrence rate with PBC than with RFT; for instance, a 2023 meta-analysis of 16 studies (*n* = 3,326) demonstrated significantly fewer 1-year recurrences with PBC (approximate OR = 0.27, *p* = 0.01) (46). Conversely, other studies have reported minimal or no long-term differences between these techniques; for example, a large single-center series (*n* = 202) found comparable recurrence-free survival for PBC and RFT during extended follow-up (47). Accordingly, although our pooled data indicate that PBC is associated with a reduced recurrence risk, we recognize the heterogeneity of findings across the broader literature. Clinicians should therefore exercise caution in interpreting this predictor, and additional comparative studies are required to elucidate the relative advantages of PBC over RFT. Third, a prolonged balloon compression duration (>120 s) was associated with an increased recurrence risk in our analysis. This counterintuitive association may be attributable to several clinical factors. One plausible explanation is selection bias by surgeons in complex cases: surgeons may prolong balloon inflation in patients with refractory or anatomically challenging TN, such that these cases inherently carry a higher baseline recurrence risk irrespective of compression duration. In essence, extended compression may serve as a marker of greater disease severity or complexity rather than a direct cause of recurrence. Additionally, exceeding the optimal compression duration may yield diminishing returns or produce uneven nerve injury. Prior studies have indicated that balloon compression durations of 60–120 s effectively relieve pain, whereas durations exceeding 120 s do not necessarily enhance outcomes and have been linked to increased recurrence (48, 49). Such discrepancies may arise from confounding variables, including balloon volume, pressure, or shape during the procedure, all of which can affect outcomes. Collectively, these hypotheses suggest that although balloon inflation exceeding 2 min correlates with recurrence, the underlying factor is likely the preferential use of prolonged compression in more challenging cases or under suboptimal conditions. We therefore emphasize the importance of individualized intraoperative decision-making and advocate for further research to establish the optimal compression duration and techniques in PBC, with the goal of maximizing long-term pain relief while minimizing recurrence.

### 5.4 Objective analysis of research limitations and biases

Application of the PROBAST tool revealed that the majority of models exhibited a high risk of bias, predominantly attributable to their reliance on retrospective data. In retrospective designs, blinding of personnel assessing predictors or outcomes is typically infeasible, thereby introducing bias. Studies employed heterogeneous definitions of “postoperative recurrence.” For instance, some utilized the BNI score, others relied on reoperation rates, and still others depended on patient self-reports. Such variability in outcome ascertainment may affect meaningful comparisons of model performance. Both the funnel plot and Egger’s test indicated substantial publication bias, with studies reporting higher AUC values being more likely to be published. This asymmetry may inflate the apparent performance of the models. Although internal validation results were frequently acceptable, external validation in independent patient cohorts was rarely performed. Consequently, the generalizability of these models to new patients remains uncertain. In summary, the high risk of bias affecting 18 of the 20 models substantially undermines confidence in their application to clinical practice. Given that nearly all models suffer from bias and inconsistent outcome definitions, estimates of recurrence risk should be interpreted with considerable caution when informing patient care. Rather than yielding readily applicable tools, the present review underscores the pressing need for future studies to construct more rigorous, prospective prediction models and to subject them to external validation. Notwithstanding these limitations, this review offers a valuable evidence synthesis in the field, consolidating findings across studies and identifying consistently reported predictors. The comprehensive overview can inform future research by elucidating key prognostic factors and highlighting priority areas for methodological refinement.

### 5.5 Reasonable prospects for future research directions

Based on this review, future research on predicting trigeminal neuralgia (TN) recurrence following surgery should prioritize the development of high-quality, clinically applicable predictive models. First, prospective multicenter studies should be conducted. A standardized definition of recurrence (e.g., the Barrow Neurological Institute [BNI] pain intensity score) should be adopted, with consistent collection of key predictor variables across all participating centers. Representative predictors include age (e.g., ≥65 years) and disease duration (e.g., >5 years). This approach would minimize heterogeneity and bias across studies. Machine learning algorithms capable of capturing nonlinear and complex interactions, such as Random Forest, are recommended for model development, as they enhance predictive performance and robustness. Finally, model evaluation should extend beyond discriminative performance to include assessment of clinical utility. Decision curve analysis should be employed to quantify net clinical benefit across a range of risk thresholds. Furthermore, the development of user-friendly interfaces, such as web-based calculators, would facilitate the integration of these models into routine clinical practice. Ultimately, the goal is to develop methodologically robust, high-evidence prediction tools that enable personalized recurrence risk stratification across surgical approaches—particularly for microvascular decompression (MVD) models exhibiting superior discriminative ability—and tailored to specific patient subgroups, thereby optimizing postoperative management.

## Data Availability

The original contributions presented in the study are included in the article/Supplementary material, further inquiries can be directed to the corresponding author.
