# Supplementary material for: Early warning of postoperative recurrence in trigeminal neuralgia: a systematic review and meta-analysis of prediction models
Source: Front Neurol. 2026 May 7;17:1772331. doi: 10.3389/fneur.2026.1772331 (PMC13189926; doi:10.3389/fneur.2026.1772331)

Study	Predictors
Tang 2025	2 factors：IL-6、TNF-α
Xia 2024	3 factors:Compressive stress and SCN8A, SCN9A gene polymorphism
Wang 2024	6 factors：Disease duration >5 years、Atypical pain、Non-pear-shaped balloon、Compression time >120s、Delayed pain disappearance、Facial numbness (protective)
Sun 2024	4 factors：Shortened trigeminal root length、Increased trigeminal atrophy、Multi-vessel compression、Severe baseline pain
Qi 2024	6 factors ：Obesity (BMI≥28 kg/m²)、Multiple sclerosis comorbidity、TN duration ≥5 years、Atypical pain、Balloon compression time >120s、V3 zone pain (protective)
Yu 2024	3factors ：Age (≥65 years)、Preoperative NRS-11 score (≥7)、Surgery type (PBC vs RFT)
Wu 2024	2 factors:original_shape_Maximum2D、Original_Shape_Elongation
Peng 2024	3 factors:Responsible vessels of non-arterial、Clinical features of atypical、CPA area ratio (healthy/affected side)>1
Li 2024	3 factors：NVC degree、Pain type、Radiomics signature (16 features)
Li 2023	2 factors：Foramen ovale length-diameter ratio、area ratio (affected/healthy side)
Wu 2023	4 factors: age, duration, branches, numbness
Wang 2023	3 factors:Numeric rating scale (NRS)、Response to neuroanalgesic drugs、Neurovascular contact on MRI
Deng 2023	3 factors:Pain grade、Type of pain、Temperature
Chen 2023	3 factors:Age (≥65 years)、Procedure (PBC vs RF)、Duration (≥5 years)
Zhao 2022	3 factors: Age≥65, Disease course≥5 years, Balloon compression surgery
Pang 2022	3 factors: Diabetes, Balloon volume, Compression time
Kourilsky 2022	6factors： longer duration of presurgical symptoms;localization of the pain along the mandibular branch of the trigeminal nerve (V3); atypical pain; diagnosis of multiple sclerosis; use of a medical device not specifically adapted for trigeminal neuralgia surgery;duration of balloon compression > 60 seconds
Zhang 2021	3 factors:Undefined responsibility vessels、Arteriovenous compression 、≥3 vessels involved
Li 2021	4 factors:Atypical pain、Disease duration ≥48 months、Previous surgical history、Preoperative BNI grade V
Shi 2020	4 factors:Younger age, Poor preoperative pain control (BNI > IV), Intraoperatively detected multivessel compression, combined compression of the superior cerebellar artery (SCA) and the petrosal vein (PV)

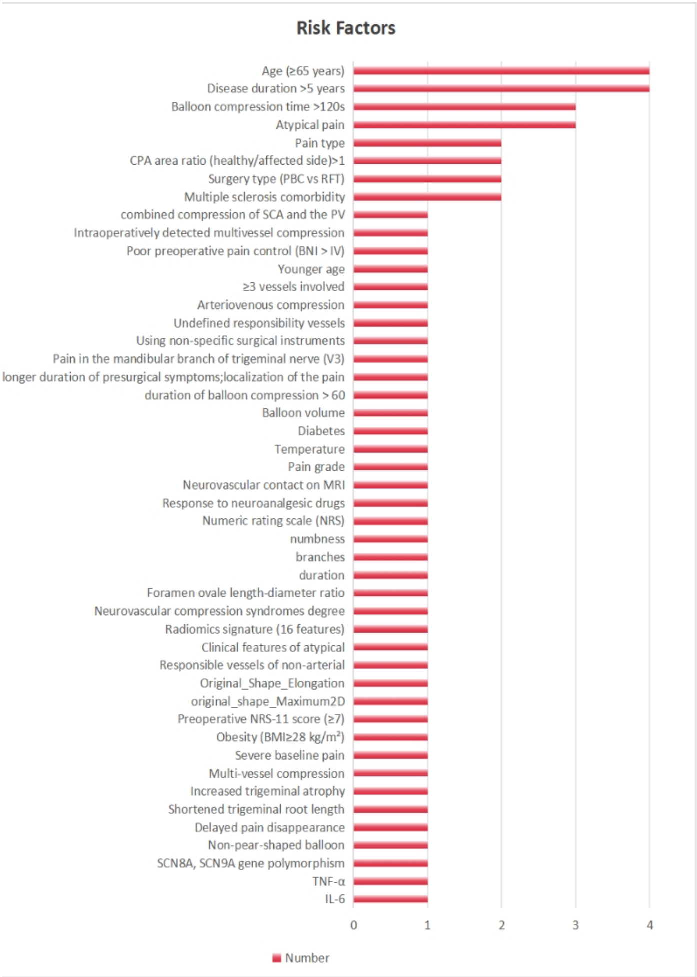

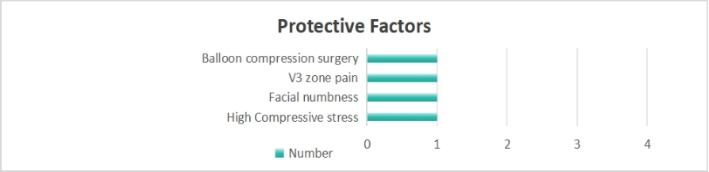

Supplement: Supplementary file 1 [file Supplementary_file_1.pdf]
