# Supplementary material for: Early warning of postoperative recurrence in trigeminal neuralgia: a systematic review and meta-analysis of prediction models
Source: Front Neurol. 2026 May 7;17:1772331. doi: 10.3389/fneur.2026.1772331 (PMC13189926; doi:10.3389/fneur.2026.1772331)
Supplement: Supplementary file 2 [file Table_1.DOC]

Supplementary Material

# 1 Search strategies

**1.1 PubMed: 53 records.**

| #1 | "prediction model"[Title/Abstract] OR "risk assessment"[Title/Abstract] OR "risk prediction"[Title/Abstract] OR "prediction tool"[Title/Abstract] OR "predictive risk model"[Title/Abstract] OR "roc curve"[Title/Abstract] OR "Discrimination"[Title/Abstract] OR "Discriminate"[Title/Abstract] OR "c-statistic"[Title/Abstract] OR "c-statistic"[Title/Abstract] OR "area under the curve"[Title/Abstract] OR "AUC"[Title/Abstract] OR "Calibration"[Title/Abstract] OR "DCA"[Title/Abstract] OR "decision curve analysis"[Title/Abstract] |
| --- | --- |
| #2 | "Trigeminal Neuralgia"[Mesh] OR "Trigeminal Neuralgia"[Title/Abstract] OR "Fothergill's Disease"[Title/Abstract] OR "Tic Douloureux"[Title/Abstract] OR TN[Title/Abstract] |
| #3 | "Recurrence"[Title/Abstract] OR "Relapse"[Title/Abstract] OR "Recurrent"[Title/Abstract] OR "Persistent Pain"[Title/Abstract] OR "Treatment Failure"[Title/Abstract] OR "Pain Recurrence"[Title/Abstract] |
| #4 | #1 AND #2 AND #3 |

**1.2 The Cochrane library: 30 records.**

| #1 | (Trigeminal Neuralgia):ti,ab,kw OR (Fothergill's Disease):ti,ab,kw OR (Tic Douloureux):ti,ab,kw OR (TN):ti,ab,kw OR (Tic Doloureux):ti,ab,kw |
| --- | --- |
| #2 | MeSH descriptor: [Trigeminal Neuralgia] explode all trees |
| #3 | #1 OR #2 |
| #4 | (postoperative):ti,ab,kw OR (post-surgical):ti,ab,kw OR (post surgery):ti,ab,kw OR (after surgery):ti,ab,kw OR (surgical):ti,ab,kw |
| #5 | (recurrence):ti,ab,kw OR (relapse):ti,ab,kw OR (recurrent):ti,ab,kw OR (recurring):ti,ab,kw OR (repeated):ti,ab,kw |
| #7 | (prediction model):ti,ab,kw OR (risk assessment):ti,ab,kw OR (risk prediction):ti,ab,kw OR (prediction tool):ti,ab,kw OR (predictive risk model):ti,ab,kw |
| #8 | (ROC Curve):ti,ab,kw OR (Discrimination):ti,ab,kw OR (Discriminate):ti,ab,kw OR (c-statistic):ti,ab,kw OR (c statistic):ti,ab,kw |
| #9 | (Area under the curve):ti,ab,kw OR (AUC):ti,ab,kw OR (Calibration):ti,ab,kw OR (DCA):ti,ab,kw OR (Decision Curve Analysis):ti,ab,kw |
| #10 | #7 OR #8 OR #9 |
| #11 | #3 AND #4 AND #5 AND #10 |

**1.3 Web of science: 281 records.**

| #1 | TS=(Trigeminal Neuralgia OR TN OR Tic Douloureux OR Facial Neuralgia OR Fifth Cranial Nerve Neuralgia OR Trifacial Neuralgia) |
| --- | --- |
| #2 | TS=(Postoperative OR Post-surgery OR After surgery OR Surgery*) |
| #3 | TS=(Recurrence OR Relapse OR Pain recurrence OR Persistent pain OR Treatment failure) |
| #4 | TS=(Prediction Model* OR Prognostic Model* OR Risk Prediction* OR Predictive Model* OR Nomogram* OR Risk Score* OR Clinical Prediction* OR Machine Learning Model* OR Regression Model* OR Scoring System*) |
| #5 | #1 AND #2 AND #3 AND #4 |

**1.4 EMBASE: 12records.**

| #1 | trigeminal neuralgia'/exp OR trigeminal neuralgia:ti,ab OR TN:ti,ab OR 'Fothergill''s disease':ti,ab OR 'tic douloureux':ti,ab |
| --- | --- |
| #2 | 'postoperative'/de OR 'postoperative period'/de OR postoperativ*:ti,ab OR post-surg*:ti,ab OR after surgery:ti,ab OR 'post procedure':ti,ab OR 'surgical treatment':ti,ab |
| #3 | 'recurrence'/de OR recurrenc*:ti,ab OR reccurent:ti,ab OR 'pain recurrence':ti,ab OR 'relapse':ti,ab OR 'persistent pain':ti,ab OR 'treatment failure':ti,ab |
| #4 | 'prediction model'/de OR 'prognostic model'/de OR 'risk prediction'/de OR 'predictive model'/de OR nomogram*:ti,ab OR 'risk score':ti,ab OR 'clinical prediction':ti,ab OR 'multivariable model':ti,ab OR 'regression model':ti,ab OR 'machine learning':ti,ab OR 'scoring system':ti,ab |
| #4 | #1 AND #2 AND #3 AND #4 |

**1.5 CNKI: 22 records.**

| #1 | 主题：三叉神经痛 + TN |
| --- | --- |
| #2 | 主题：术后 |
| #3 | 主题：复发+反复发作+疼痛复发+治疗效果不佳 |
| #4 | 篇关摘：预测模型 + 风险预测 + 风险模型 + c统计量 + AUC + ROC + ROC曲线 + 曲线下面积 + DCA曲线 + DCA + 校准 + 区分度 |
| #5 | #1 AND #2 AND #3 AND #4 |

**1.6 Wanfang: 57 records.**

| #1 | 题目或关键词=(三叉神经痛 OR TN) |
| --- | --- |
| #2 | 题目或关键词=(术后) |
| #3 | 题目或关键词=(复发 OR 再发 OR 疼痛复发 OR治疗效果不佳) |
| #4 | 题目或关键词=(预测模型 OR 风险预测 OR 风险模型 OR c统计量 OR AUC OR ROC OR ROC曲线 OR 曲线下面积 OR DCA曲线 OR DCA OR 校准 OR 区分度) |
| #5 | #1 AND #2 AND #3 AND #4 |

**1.7 VIP: 3 records.**

| #1 | 题目或关键词=(三叉神经痛 OR TN) |
| --- | --- |
| #2 | 题目或关键词=(术后) |
| #3 | 题目或关键词=(复发 OR 再发 OR 疼痛复发 OR治疗效果不佳) |
| #4 | 题目或关键词=(预测模型 OR 风险预测 OR 风险模型 OR c统计量 OR AUC OR ROC OR ROC曲线 OR 曲线下面积 OR DCA曲线 OR DCA OR 校准 OR 区分度) |
| #5 | #1 AND #2 AND #3 AND #4 |

**1.8 SinoMed: 13 records.**

| #1 | "三叉神经痛"[常用字段:智能] OR "TN"[常用字段:智能] |
| --- | --- |
| #2 | "术后"[常用字段:智能] |
| #3 | "复发"[常用字段:智能] OR "再发"[常用字段:智能] OR "疼痛复发"[常用字段:智能] OR "治疗效果不佳"[常用字段:智能] |
| #4 | "预测模型"[常用字段:智能] OR "风险预测"[常用字段:智能] OR "风险模型"[常用字段:智能] OR "c统计量"[常用字段:智能] OR "AUC"[常用字段:智能] OR "ROC"[常用字段:智能] OR "ROC曲线"[常用字段:智能] OR "曲线下面积"[常用字段:智能] OR "DCA曲线"[常用字段:智能] OR "DCA"[常用字段:智能] OR "校准"[常用字段:智能] OR "区分度"[常用字段:智能] |
| #5 | #1 AND #2 AND #3 AND #4 |
