# Supplementary material for: Early warning of postoperative recurrence in trigeminal neuralgia: a systematic review and meta-analysis of prediction models
Source: Front Neurol. 2026 May 7;17:1772331. doi: 10.3389/fneur.2026.1772331 (PMC13189926; doi:10.3389/fneur.2026.1772331)
Supplement: Supplementary file 3 [file Table_2.DOC]

Supplementary Material

Supplementary Material 2. Characteristics of Prediction Models.

| **Study** | **No. of relapses (recurrence rate)** | **EPV** | **Development methodology** | **Sample size** | | **Validation Methodology** | **Model presentation** | **Calibration measure** | **Discrimination measure** | | **DCA measure** | **TRIPOD Levels^a^** |
| --- | --- | --- | --- | --- | --- | --- | --- | --- | --- | --- | --- | --- |
|  |  |  |  | **Modeling** | **Validation** |  |  |  | **Construction set** | **Validation set** |  |  |
| Tang 2025 | 24（12.83%） | 12.00 | Logistic Regression | NR | NR | NR | NR | NR | IL-6：0.827 TNF-α：0.812 Combined：0.921 |  | NR | 1a |
| Xia 2024 | Exposed group: 11 (5.29%) Non-exposed group: 25 (13.37%) | 12.00 | Cox regression | NR | NR | NR | Nomogram | Hosmer-Lemeshow χ²=2.449, P=0.964 | 0.813 (0.728-0.898) |  | Best applicability when threshold 0.03-0.55 | 1a |
| Wang 2024 | Modeling group: 108 (34.1%) Validation group: 43 (32.8%) | 25.17 | Logistic regression； Random forest； XGBoost | 131 |  | External validation | Nomogram | NR | RF:0.824(0.774-0.873); LR:0.810; XGBoost:0.816 | RF:0.835(0.763-0.892); LR:0.817; XGBoost:0.822 | NR | 3 |
| Sun 2024 | 56 (18.9%) | 14.00 | Logistic Regression | NR | NR | NR | Formula | NR | Trigeminal root length: 0.553（0.469-0.637） Trigeminal atrophy: 0.750（0.669-0.832） Combined: 0.861（0.803-0.919） |  | NR | 1a |
| Qi 2024 | Training set: 95 (56.9%) Test set: 47 (50.0%) | 23.67 | Logistic Regression | 167 | 94 | Internal validation | Formula | Goodness-of-fit test (χ²=0.716, P<0.05) | 0.789 | 0.792 | NR | 2b |
| Yu 2024 | 55 (42.0%) | 18.33 | Logistic Regression | 92 | 39 | Internal validation | Nomogram、Formula | Hosmer-Lemeshow test: P>0.05 (good fit) | 0.890 (0.818-0.961) | 0.857 (0.748-0.965) | Net benefit higher at threshold probability 0-0.990 | 2a |
| Wu 2024 | 16(13.68%) | 8.00 | Machine Learning:Random Forest 、Logistic Regression | NR | NR | k-fold cross-validation (k=5) | Nomogram | The calibration curve shows the consistency of satisfactory. | original_shape_Maximum2D：0.812 Original_Shape_Elongation：0.874 combined：0.872 |  | The decision-making curve shows that the pillar diagram of the risk of effective results increases more benefits than the full or no scene. | 1b |
| Peng 2024 | 14 (11.3%) | 4.67 | Logistic Regression | 87 | 37 | Internal validation | Nomogram | NR | 0.91 | 0.859 | Decision Curve DCA: Reported significant net benefit | 2b |
| Li 2024 | Development cohort: 63 (22.6%) Test cohort: 49 (27.8%) | 37.33 | Cox regression | 279 | 176 | External validation | Nomogram、Formula | Good calibration (Hosmer-Lemeshow test p=0.718 development, p=0.214 test) | 0.865 | 0.834 | Nomogram provided largest net benefit across most threshold probabilities | 3 |
| Li 2023 | 10(16.7%) | 5.00 | Logistic Regression | NR | NR | NR | NR | NR | Length-diameter ratio: 0.792 (0.628-0.956) Area ratio: 0.766 (0.591-0.941) |  | NR | 1a |
| Wu 2023 | 53(27.0%） | 13.25 | random forest | 137 | 59 | Internal validation | Nomogram | Good fit in both training and test groups (Hosmer-Lemeshow test P>0.05) | 0.726 (0.626-0.827) | 0.611(0.441-0.780) | Net benefit high at threshold probability 0-0.9 | 2b |
| Wang 2023 | 13(23.21%) | 4.33 | Logistic Regression | NR | NR | Internal validation | Online dynamic nomogram | Good fit shown by calibration curve (P=1 in Hosmer-Lemeshow test) | 0.973 (0.938-1.000) |  | Model showed net benefit when threshold probability was between 0-100% | 1b |
| Deng 2023 | 61(43.88%） | 20.33 | Cox Regression | NR | NR | Internal validation | Nomogram | Calibration curve showed: Overestimated risk when observed probability <90% Underestimated risk when observed probability >90% Brier score: ~0.14 | 6 months: 0.787 12 months: 0.705 18 months: 0.672 24 months: 0.689 |  | Net benefit higher in certain probability range (no specific values given) | 1a |
| Chen 2023 | 27(17.4%) | 9.00 | Logistic Regression | 108 | 47 | Internal validation | Nomogram | Good fit in both training and test groups (Hosmer-Lemeshow test P>0.05) | 0.749 (0.618- 0.879) | 0.793(0.584- 0.980) | Net benefit higher at threshold probability 0-0.486 | 2b |
| Zhao 2022 | 17 (13.8%) | 5.67 | Logistic regression | NR | NR | Internal validation | Nomogram | Good fit (Brier score not provided, Hosmer-Lemeshow P=0.810) | 0.818 (0.733-0.903) |  | NR | 1a |
| Pang 2022 | 24 (17.91%) | 8.00 | Logistic regression | NR | NR | Internal validation | Nomogram | calibration curve shows good consistency | 0.991 (0.978-1.000) |  | NR | 1b |
| Kourilsky 2022 | 77 (58.8%) | 12.83 | Cox regression | NR | NR | Internal validation | Nomogram | NR | 0.77 |  | NR | 1a |
| Zhang 2021 | Modeling group: 35/191 (18.32%) Validation group: 10/58 (17.24%) | 15.00 | Logistic regression | 200 | 60 | Internal validation | Nomogram | Good (shown by calibration curve) | 0.917 (0.854-0.949) |  | NR | 2b |
| Li 2021 | 35 (8.0%) | 8.75 | Logistic regression | NR | NR | Internal validation | NR | NR | 0.729 (0.701-0.913) |  | NR | 1a |
| Shi 2020 | 51(27.7%) | 12.75 | Logistic regression | NR | NR | NR | NR | NR | Younger age: 0.774(0.68–0.86)​Multivessel compression: 0.871(0.79–0.95)​​ ​Preoperative BNI > IV: 0.858(0.79–0.93)  Combined SCA +PV:0.812(0.81–0.91)All three combined:0.968 (0.94–0.99)​ |  | NR | 1a |

a Transparent Reporting of a multivariable prediction model for Individual Prognosis Or Diagnosis (TRIPOD) levels: 1a=development only; 1b=development and validation using resampling; 2a=random split sample development and validation; 2b=non-random split sample development and validation; 3=development and validation using separate data; 4=validation only

EPV (events per variable) indicates the number of events per independent variable.AUC indicates area under the ROC curve; CI indicates 95% confidence interval; H-L indicates Hosmer-Lemeshow goodness-of-fit test; LR denotes logistic regression; XGBoost denotes extreme gradient enhancement; RF denotes random forest; — denotes missing information；IL-6 indicates Interleukin-6；TNF-αindicates Tumor Necrosis Factor-alpha；SCA denotes superior cerebellar artery；PV denotes petrosal vein.
